# Dimensionality Reduction by Supervised Neighbor Embedding Using Laplacian Search

**DOI:** 10.1155/2014/594379

**Published:** 2014-05-21

**Authors:** Jianwei Zheng, Hangke Zhang, Carlo Cattani, Wanliang Wang

**Affiliations:** ^1^School of Computer Science and Technology, Zhejiang University of Technology, Hangzhou 310023, China; ^2^Department of Mathematics, University of Salerno, Via Ponte Don Melillo, 84084 Fisciano, Italy

## Abstract

Dimensionality reduction is an important issue for numerous applications including biomedical images analysis and living system analysis. Neighbor embedding, those representing the global and local structure as well as dealing with multiple manifolds, such as the elastic embedding techniques, can go beyond traditional dimensionality reduction methods and find better optima. Nevertheless, existing neighbor embedding algorithms can not be directly applied in classification as suffering from several problems: (1) high computational complexity, (2) nonparametric mappings, and (3) lack of class labels information. We propose a supervised neighbor embedding called discriminative elastic embedding (DEE) which integrates linear projection matrix and class labels into the final objective function. In addition, we present the Laplacian search direction for fast convergence. DEE is evaluated in three aspects: embedding visualization, training efficiency, and classification performance. Experimental results on several benchmark databases present that the proposed DEE exhibits a supervised dimensionality reduction approach which not only has strong pattern revealing capability, but also brings computational advantages over standard gradient based methods.

## 1. Introduction

The classification of high-dimensional data, such as biological characteristic sequences, high-definite images, and gene expressions, remains a difficult task [1]. The main challenges that these high-dimensional data pose include inferior outcome performance, tremendous storage requirements, and high computational complexity. Dimensionality reduction (DR) [2], as the core research topic in subspace learning community, is the well-acknowledged solution for this curse of dimensionality problem. For the classification tasks, the goal of DR focuses on constructing a low-dimensional representation of data in order to achieve better discrimination and accelerate the subsequent processing. In this realm, very straightforward algorithms are dominated, as the computational complexity of advanced DR techniques proposed is too high.

Fisher discriminant analysis (FDA) [3] and its variants [4, 5], which incorporate the class labels information and aim at the preservation of classification accuracy in the embedded subspace, are the mostly adopted DR techniques. FDA amplifies the between-class scatter and simultaneously shrinks the within-class scatter in subspace for the purpose of desirable separability. Recently, LFDA [6], MMDA [7], DCV [8], and MMPA [9] markedly improve the performance of FDA by solving different kinds of existing thorny issues. LFDA adds the locality preservation property to the Fisher criterion, which preserves the multimodal structure. MMDA presents a novel criterion that straightly maximizes the minimum pairwise distances of the whole classes for better between-class separability. DCV circumvents the “small sample size” problem by using two different methods, the within-class scatter matrix and the Gram-Schmidt orthogonalization procedure, to obtain the discriminative common vectors. MMPA takes into account both intraclass and interclass geometries and also possesses the orthogonality property for the projection matrix. Broadly speaking, all these methods have a unique solution computed by a generalized eigensolver and exhibit acceptable performance on most data, but they may be suboptimal for the data with nonuniform density or multiple manifolds.

To deal with more complex structural data, especially in biomedical applications [10–12], a batch of novel DR techniques [13–19] based on stochastic neighbor embedding (SNE) [13] absorbs a lot of interest. In contrast with the FDA-type techniques, those consider only the original high-dimensional space for constructing the objective function. SNE matches similarities which are achieved both from the high-dimensional and low-dimensional spaces. Furthermore, *t*-SNE [14] extends SNE with symmetric similarities and by using student's *t*-distribution in low-dimensional space. Symmetric SNE [15] explains why the heavy-tailed distribution and the symmetric similarity form in *t*-SNE lead to better performance. NeRV [16] uses the “dual” Kullback-Leibler (KL) divergence for well-content DR results in information retrieval perspective. Lee et al. [17] adopted a scaled version of the generalized Jensen-Shannon divergence that better preserves small K-ary neighborhoods. Bunte et al. [18] analyzed the visualization performance of SNE with arbitrary divergences and claimed that KL divergence is the most acceptable. In terms of visualization results, all these techniques outperform most of the past techniques. However, the reasons of this well behavior remain obscure. Lee and Verleysen [20] investigated the role played by the specific similarities and identified that appropriate normalization with property of shift invariance is the main cause of the admirable performance. However, Carreira-Perpiñán [21] revealed the fundamental relation between SNE and the Laplacian eigenmaps [22] method and proposed a new DR method, the elastic embedding (EE), that can be seen as learning both the coordinates and the affinities between data points without the shift invariance property.

EE is more efficient and robust than SNE, *t*-SNE, and NeRV. Even so, it cannot be directly applied in classification tasks because of the unideal discrimination ability, the out-of-sample problem, and the high computational complexity in some large-scale classification tasks [23, 24]. Many researchers have been dedicated to solve these drawbacks. Venna et al. [16] proposed supervised NeRV with better discrimination capability by complex locally linear functions. Bunte et al. [25] presented a general framework for a variety of nonparametric DR techniques and then extended them to parametric mapping by means of optimization. Gisbrecht et al. [26] used only a fraction of whole samples for training the DR model in interactive settings. Yang et al. [27] and Maaten [28] simultaneously adopted the Barnes-Hut tree and proposed a generic approximated optimization technique which reduces the neighbor embedding optimization cost from *O*(*N* ^2^) to *O*(*N*log⁡*N*).

Inspired by these works, we proposed a linear supervised DR technique called discriminative EE (DEE) for classification. To be specific, the linear projection matrix is introduced to solve the out-of-sample problem similarly as in [29]. The class labels information is involved in the construction of objective function as MMPA. We search for a reasonable direction in the iterative processing to solve our model by gradient-based method. The remainder of this paper is structured as follows.  Section 2 provides a brief view of related works.  Section 3 describes the objective function and the search direction of our proposed DEE.  Section 4 gathers the experimental results. Finally, Section 5 draws the conclusions and sketches some future works.

## 2. Fundamental Contributions

Even though there were numerous previously studied algorithms in the context of embedding high-dimensional data for visualization or classification, we focus here only on a few approaches that were recently proposed and that we will use to compare our evaluations against them. The involved techniques include the elastic embedding (EE), the discriminative stochastic neighbor embedding (DSNE) [30], and the min-max projection analysis (MMPA). Let **x**_*i*_ ∈ *R* ^*D*^  (*i* = 1,2,…, *N*) be *D*-dimensional samples, *l*_*i*_ ∈ (1,2,…, *C*) be corresponding class labels and **X** = {**x**_1_, **x**_2_,…, **x**_*N*_} be the matrix of all samples, where *N* is samples size and *C* is the classes size. The nonlinear embedding approaches proceed to look for the subspace matrix **Y** ^*d*×*N*^, whose column vectors **y**_*i*_ ∈ *R* ^*d*^ are coordinates of pixel maps, where* d* is the subspace dimension. On the other hand, the goal of the usual linear embedding techniques is to learn a projection matrix **A** ^*d*×*D*^, which is further used to compute the embedding coordinate **Y** = **A** **X**.

### 2.1. The Elastic Embedding

The elastic embedding (EE) technique, which is nonlinear and unsupervised, is an extension of SNE-type algorithm. The objective function of EE is defined as

(1) E(Y)=∑n,m=1Nωnm+||yn−ym||2+λ∑n,m=1Nωnm−exp⁡⁡(−||yn−ym||2),

where *ω*_*nm*_^+^ = exp⁡(−||**x**_*n*_−**x**_*m*_||^2^/2*σ* ^2^) and *ω*_*nm*_^−^ = ||**x**_*n*_−**x**_*m*_||^2^, ∀*n* ≠ *m*, with *ω*_*nm*_^+^ = *ω*_*nm*_^−^ = 0. The left (+) term in (1), called as attractive term, preserves local distances as the Laplacian eigenmaps [22]. The right (−) term in (1), called as repulsive term, preserves global distances in a plainer way more than the traditional SNE algorithm. *λ* is a tunable parameter for trading off both the attractive and the repulsive terms.

The gradient of *E*(**Y**) in (1) is then computed as

(2) $$\begin{array}{c} G\left(Y\right)=\frac{\partial E}{\partial Y}=4Y\left(L^{+}-\lambda {\tilde{L}}^{-}\right), \end{array}$$

where the authors defined the affinities

(3) $$\begin{array}{c} {\tilde{\omega }}_{nm}^{-}=\omega_{nm}^{-}\exp\left(-{||y_{n}-y_{m}||}^{2}\right) \end{array}$$

and the graph Laplacians **L** = **D** − **W** in the common way. **D** = diag⁡(∑_*n*=1_^*N*^ *ω*_*nm*_) is the degree matrix. After the gradient is obtained, EE uses the fixed-point (FP) diagonal iteration scheme to achieve global and fast convergence. First, the gradient is split as

(4) $$\begin{array}{c} \nabla E=4Y\left(D^{+}+\left(L^{+}-\lambda {\tilde{L}}^{-}-D^{+}\right)\right)=0; \end{array}$$

then, a search direction is derived as **Y**(**D** ^+^ − **L** ^+^ − *λ* **L** ^−^)(**D** ^+^)^−1^ − **Y**.

Both the objective function and the gradient for EE are intuitively clearer and less nonlinear than other SNE-type algorithms since EE avoids the cumbrous log-sum term. Moreover, the FP strategy results in fewer local optima. However, EE is still nonlinear, so the embedding for the out-of-sample input is inefficient. Furthermore, EE neglects the use of the class labels, which makes EE suboptimal for classification.

### 2.2. Discriminative Stochastic Neighbor Embedding

The discriminative stochastic neighbor embedding (DSNE) technique, which is linear and supervised, is an extension of *t*-SNE algorithm. For each input data **x**_*i*_ and each potential neighbor **x**_*j*_ within the same class or not, the probability *p*_*ij*_ that **x**_*i*_ selects **x**_*j*_ as its neighbor is

(5) $$\begin{array}{c} p_{ij}=\{\begin{array}{cc} \frac{\exp\left(-{||x_{i}-x_{j}||}^{2}/2\sigma_{}^{2}\right)}{\sum_{l_{k}=l_{l}}\exp\left(-{||x_{k}-x_{l}||}^{2}/2\sigma_{}^{2}\right)} & \text{if}\;l_{i}=l_{j}\\ \frac{\exp\left(-{||x_{i}-x_{j}||}^{2}/2\sigma_{}^{2}\right)}{\sum_{l_{k}\neq l_{m}}\exp\left(-{||x_{k}-x_{m}||}^{2}/2\sigma_{}^{2}\right)} & \text{else}, \end{array} \end{array}$$

where *σ* is a regularization parameter which is set manually. For the embedded samples **Y** = **A** **X**, a heavy-tailed Student's *t*-distribution with one degree of freedom for neighbors is made, so that the induced embedded probability *q*_*ij*_ that **y**_*i*_ selects **y**_*j*_ as its intraclass or interclass neighbors is

(6) $$\begin{array}{c} q_{ij}=\{\begin{array}{cc} \frac{{\left(1+{\left(x_{i}-x_{j}\right)}^{T}A^{T}A\left(x_{i}-x_{j}\right)\right)}^{-1}}{\sum_{l_{k}=l_{l}}{\left(1+{\left(x_{k}-x_{l}\right)}^{T}A^{T}A\left(x_{k}-x_{l}\right)\right)}^{-1}} & \text{if}\;l_{i}=l_{j}\\ \frac{{\left(1+{\left(x_{i}-x_{j}\right)}^{T}A^{T}A\left(x_{i}-x_{j}\right)\right)}^{-1}}{\sum_{l_{k}\neq l_{m}}{\left(1+{\left(x_{k}-x_{m}\right)}^{T}A^{T}A\left(x_{k}-x_{m}\right)\right)}^{-1}} & \text{else}. \end{array} \end{array}$$

The aim of DSNE is to place close together intraclass samples and place far apart interclass samples. This is achieved by minimizing the objective function, which is the sum of KL divergences between *p*_*ij*_ and *q*_*ij*_ with consideration of the class labels

(7) $$\begin{array}{c} E\left(A\right)=\underset{l_{i}=l_{j}}{\sum }p_{ij}\log\frac{p_{ij}}{q_{ij}}+\underset{l_{i}\neq l_{k}}{\sum }p_{ik}\log\frac{{p_{i}}_{k}}{q_{ik}}. \end{array}$$

The gradient of *E*(**A**) in (7) can be obtained as

(8) $$\begin{array}{c} \frac{\partial E}{\partial A}=4A\left\{X\left(L^{\text{intra}}+L^{\text{inter}}\right)X^{T}\right\}, \end{array}$$

where **L** ^intra^ and **L** ^inter^ are the Laplacian matrices for intraclass samples and interclass samples, respectively. DSNE introduces explicit projection matrix and labels information to make it suitable for classification tasks. However, the cumbersome log-sum term and the tedious conjugate gradient training method make DSNE converge slowly.

### 2.3. Min-Max Projection Analysis

The min-max projection analysis (MMPA) is another recently proposed linear supervised dimension reduction technique. MMPA combines the main advantages of block optimization and whole alignment strategy [31]. It also incorporates a desirable property from marginal Fisher analysis [32], that is, pulling together the far apart within class neighbors as close as possible, as well as placing away the neighbors having different labels as far as possible. The combination of these properties leads to a technique that is qualified for online input stream data and has desirable discrimination capability. The projection matrix **A** derived from MMPA is a result of solving the following objective function:

(9) $$\begin{array}{c} A=\arg\underset{A\in R^{d\times D}}{\min}\mathrm{tr}\left(\frac{AXL^{\text{intra}}X^{T}A^{T}}{AXL^{\text{inter}}X^{T}A^{T}}\right). \end{array}$$

By resolving the generalized eigenvalue problem in (9), MMPA gets a closed form solution without any iteration process, which is closely related to the classical dimension reduction algorithms such as DCV and LFDA. All these techniques present efficient computation cost. However, they always present crowding problem that leads to cluttered subspace visualization.

## 3. Discriminative Elastic Embedding

In this section, we depict the DEE technique that focuses on exploring an explicit mapping, presenting a large disjoint interclass region and achieving a faster convergence. We begin with an introduction of the embedding formulation.

### 3.1. Formulation

As mentioned in Section 2, the eigenmap-type algorithms such as MMPA adopt simple affinity function for constructing direct generalization eigenvalue problems, which is sensitive to noise and results in crowded embedded subspace. DSNE can go beyond the spectral techniques and find better optima, exhibiting large gaps between different classes as well as dealing with multiple manifolds. However, the optimization of DSNE is costly and apt to local optima. In addition, our understanding of these SNE-type algorithms is limited to an intuitive interpretation of their cost function. EE furthers our understanding by deriving the explicit relation between SNE and Laplacian eigenmaps. Moreover, EE adopts the simpler unnormalized model for more efficient and robust optimization. The objective function of EE is formed by a local distance term and a global distance term to represent better global and local embedding structure. However, the purpose of this paper is to enlarge the disjoint region for the different classes. We resolve this problem by introducing the class labels to both the attractive affinity weights and the repulsive affinity weights similar to MMPA:

(10) $$\begin{array}{c} \omega_{ij}^{+}=\{\begin{array}{cc} \exp\left(\frac{-{||x_{i}-x_{j}||}^{2}}{2\sigma^{2}}\right), & \text{if}\;l_{i}=l_{j}\\ 0, & \;\text{else} \end{array}\; \end{array}$$

(11) $$\begin{array}{c} \omega_{ij}^{-}=\{\begin{array}{cc} {||x_{i}-x_{j}||}^{2}, & \text{if}\;l_{i}\neq l_{j}\\ 0, & \text{else}. \end{array} \end{array}$$

In addition, we adopt the explicit projection matrix **A** to make EE linear and avoid the out-of-sample problem. That is, in (1), we replace **y** as **A** **x** to make it become

(12) E(A)=∑n,m=1Nωnm+||Axn−Axm||2+λ∑n,m=1Nωnm−exp⁡⁡(−||Axn−Axm||2)=∑n,m=1Nωnm+(xn−xm)TATA(xn−xm)+λ∑n,m=1Nωnm−exp⁡(−(xn−xm)TATA(xn−xm)).

Equation (12) is chosen here as our objective function. We call the model as discriminative elastic embedding (DEE). In next sections, we will present the optimization strategy for the optimal projection matrix **A**.

### 3.2. The Fixed-Point Search Direction

The cost function in (12) characterizes the desired embedding: objects of intraclass samples are encouraged to embed nearby, but objects of interclass samples are encouraged to embed far away. However, this equation is nonconvex, and there is no closed-form solution. Some gradient based methods such as gradient descent, conjugate gradients, and multiplicative updates [33] are used for the existing SNE-type algorithms. These are all reported as very slow and with tiny steps. The fixed-point iteration strategy used in EE works much better, so we introduce this FP method into our DEE technique in this section. The gradient of DEE is obtained as

(13) ∂E∂A=2A∑n,m=1N(ωnm+−λωnm−exp⁡(−xnmTATAxnm))xnmxnmT=4A∑n,m=1N(ωnm)(xnxnT−xnxmT)=4AX(D+−W+−λD−+λW−)XT=4AX(L+−λL−)XT=4AXLXT,

where we replace **x**_*nm*_ = **x**_*n*_ − **x**_*m*_ and *ω*_*nm*_ = *ω*_*nm*_^+^ − *λω*_*nm*_^−^exp⁡(−||**y**_*n*_−**y**_*m*_||^2^) for brevity. From the stationary point equation of the gradient (13), we can split ∂*E*/∂**A** into two parts as

(14) $$\begin{array}{c} \frac{\partial E}{\partial A}=4AX\left(D^{+}+L-D^{+}\right)X^{T}=0, \end{array}$$

where **D** ^+^ is symmetric, positive, and definite and (**L** − **D** ^+^) is symmetric. Then, we can get the FP iteration scheme **A** ← **A** **X**(**D** ^+^ − **L**)**X** ^*T*^(**X** **D** ^+^ **X** ^*T*^)^−1^, which further implies the FP search direction Δ_FP_ = **A** **X**(**D** ^+^ − **L**)**X** ^*T*^(**X** **D** ^+^ **X** ^*T*^)^−1^ − **A**. The objective function *E* will be decreased and converged to a stationary point along with the FP direction by a line search **A** ← **A** + *α*Δ_FP_ satisfying the Wolfe conditions for *α* > 0 [34]. The main cost of each FP iteration is dominated by the gradient equation (13) which is *O*(2*dDN* + *dN* ^2^).

### 3.3. The Laplacian Search Direction

Our goal in this section is to present a search direction that can lead to fast and global convergence. There are two common ways for achieving this objective. One is to speed up the computation in the gradient based iteration scheme. The other is to achieve the optimal objective value with as a few iterations as possible. The intuitive method for speeding up the computation is to reduce the samples size. This obvious approach of subsampling always produces inferior results. In [27, 28], the authors simultaneously adopted the Barns-Hut tree to approximate the SNE-type gradients, which leads to substantial computational advantages over existing neighbor embedding techniques. However, this Barns-Hut tree strategy requires sufficient training samples for maintaining preferable performance. Moreover, the Barns-Hut tree based neighbor embedding methods can only be applied for embedding data in two or three dimensions subject to the tree size. In conclusion, we turn to explore the best search direction for less iteration.

From the numerical optimization theory [34], we repeat the line search method as

(15) Ak+1=Ak+αkΔk,HkΔk=−gk,

where Δ_*k*_ is the chosen search directions, **g**_*k*_ is the gradient of the objective function, **H**_*k*_ is a positive definite matrix ensuring the descent direction Δ_*k*_^*T*^ **g**_*k*_ < 0, and *α*_*k*_ > 0 satisfies the Wolfe conditions. Our purpose here is to select a desirable matrix **H**_*k*_ ranges from** I** to the Hessian matrix obtained as

(16) $$\begin{array}{c} \frac{\partial^{2}E}{\partial A^{2}}=4\left(XLX^{T}\right)⊗I_{d}+4\left(I_{D}⊗A\right)\frac{\partial XLX^{T}}{\partial A}, \end{array}$$

where **I**_*d*_ is the *d* × *d* identity matrix. When **H**_*k*_ is selected as the identity matrix** I**, the optimization is refined as gradient descent, which is very slow for convergence. At the other extreme, when the Hessian is used, the optimization is termed as Newton's method, which consumes too much computation each iteration. Our intuitive principle is to employ as much Hessian info as possible that leads to an efficient solution of the Δ_*k*_ linear equation (15) (e.g., sparse and constant **H**_*k*_). We further split (16) as

(17) ∂2E∂A2=4(XL+XT)⊗Id−4λ(XL−XT)⊗Id−4λ(ID⊗A)∂XL−XT∂A.

Since **L** ^−^ is closely related to the variable **A**, the second part and the third part of (17) need recomputation each iteration. The first part is constant and it only needs be computed once in the first iteration. Moreover, since the entries in **W** ^+^ are symmetric and nonnegative from (10), the term **X** **L** ^+^ **X** ^*T*^ is symmetric, positive, and semidefinite, and we can add a small *μ* **I** to it for achieving a positive definite matrix. In conclusion, the attractive Hessian 4(**X** **L** ^+^ **X** ^*T*^)**I**_*d*_ constructs our final search direction which is the desirable compromise of fast convergence and efficient calculation. Since this direction is mainly related to the attractive Laplacian **L** ^+^, we call it as the Laplacian direction (LD). Note that to avoid the direct calculation of **H**_*k*_Δ_*k*_ = −**g**_*k*_ which costs *O*(*N* ^3^ × *D*) we can firstly achieve the Cholesky factor** R** of **H**_*k*_ and then use two backsolves **R** ^*T*^(**R**Δ_*k*_) = −**g**_*k*_ for the Laplacian direction Δ_*k*_. The cost of Cholesky decomposition is in *O*(*D* ^3^/3) and it needs only to be computed once. The cost of two backsolves is in *O*(*D* ^2^ *d*). We find the Laplacian direction works surprisingly well with more less iteration times.

## 4. Experiments and Results

We evaluate the performance of the proposed algorithm in this section. First, four methods are compared for DEE: gradient descent (GD), used in SNE; conjugate gradients (CG), used in *t*-SNE; fixed-point (FP), used in EE; and the Laplacian direction (LD), presented in this paper. Afterwards, we demonstrate the effectiveness of DEE in clustering visualization compared with some classical algorithms such as *t*-SNE, DSNE, and EE. Finally, we present the experimental results on image classification. Four datasets are used for evaluation: the COIL20 images database, the ORL faces database, the Yale faces database, and the USPS handwritten digits database.

### 4.1. Datasets

**Figure 1:**
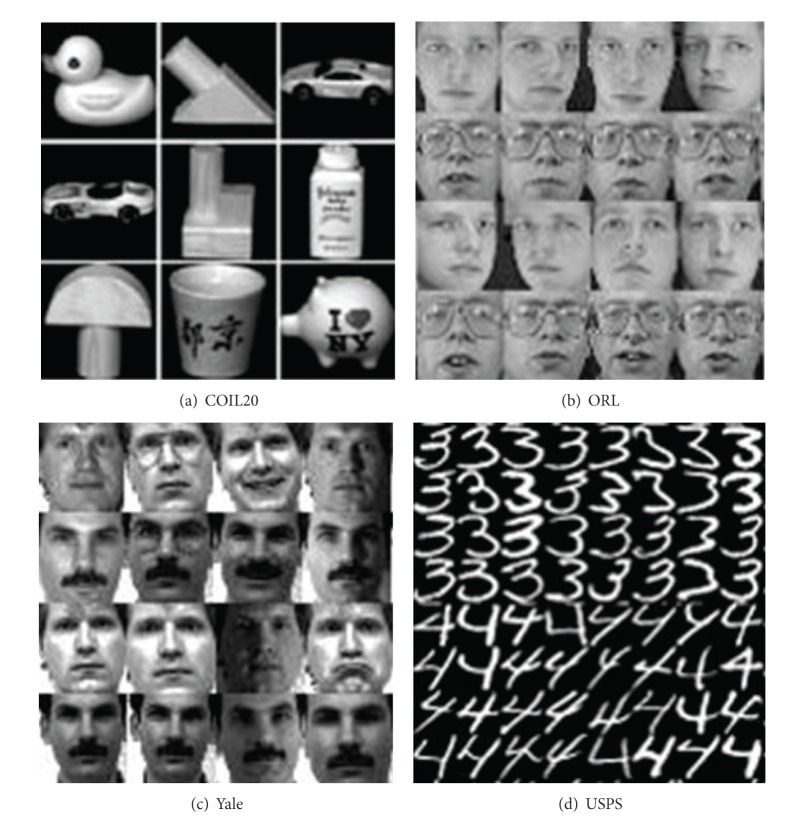
Some example images from four datasets: (a) COIL20, (b) ORL, (c) Yale, and (d) USPS.

The COIL20 database contains 20 objects. The images of each object were taken 5 degrees apart as the object is rotated on a turntable and each object has 72 images. The size of each image is 40 × 40 pixels, with 256 grey levels per pixel. The ORL face database consists of a total of 400 face images from 40 people (10 samples per person). For every subject, the images were taken at different times, varying the lighting, facial expressions (open/closed eyes, smiling/not smiling), and facial details (glassed/not glassed). All the images were taken against a dark homogeneous background with the subjects in an upright, front position (with tolerance for some side movements). The Yale database consists of 165 face images of 15 individuals. There are 11 images per subject, one per different facial expression or configuration: center-light, with glasses, happy, left-light, no glasses, normal, right-light, sad, sleepy, surprised, and wink. We preprocessed these original images by aligning transformation and scaling transformation so that the two eyes were aligned at the same position. Then, the facial areas were cropped into the resulting images. In our experiments, each image in ORL and Yale database was manually cropped and resized to 32 × 32 pixels. USPS handwritten digits dataset includes 10 digit characters and 1100 samples in total. The data format is of 16 × 16 pixels.  Figure 1 shows some example images from the four datasets.

### 4.2. The Evaluation of Different Training Methods

Many different training methods have been applied for solving the SNE-type embedding algorithms. We have implemented the following four methods for optimizing DEE model to be compared with the Laplacian direction method described in Section 3: gradient descent (GD), originally used for SNE; conjugate gradient (CG), originally used for *t*-SNE; and fixed-point iteration, originally used for symmetric SNE and EE. There are several parameter values that require the user to set for all the three implemented methods. Commonly, there is little clue on which parameter values are the most appropriate. This is the main reason why LD method, which has no parameters to be set, is our preferred choice. We set most of the parameters to be the same as [13, 14, 21]. The maximum iterations were set 1000 constantly and the ultimate convergence condition was set to be 1*e* − 3. For the first three datasets, COIL20, ORL, and Yale, we used all the samples as the input data. And for avoiding clutter, we randomly selected sixty samples for every class as the input data for the USPS handwritten digits dataset.

**Figure 2:**
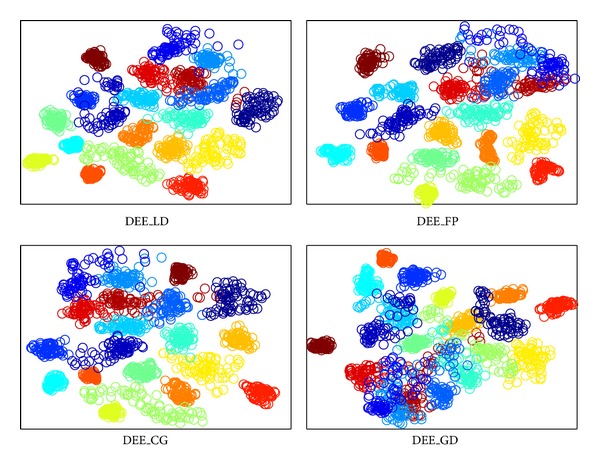
The clustering visualization for COIL20 data set with different training methods.

**Figure 3:**
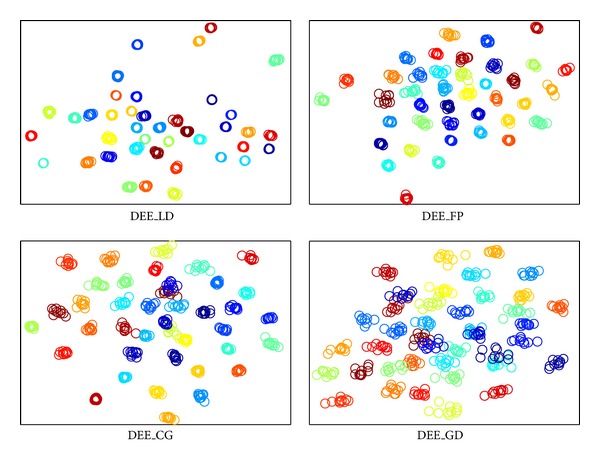
The clustering visualization for ORL data set with different training methods.

**Figure 4:**
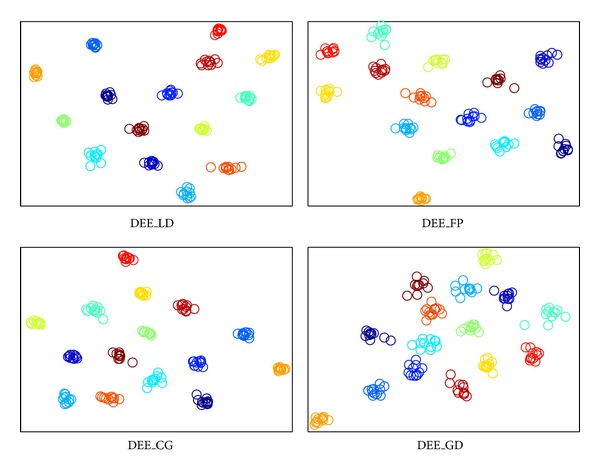
The clustering visualization for Yale data set with different training methods.

**Figure 5:**
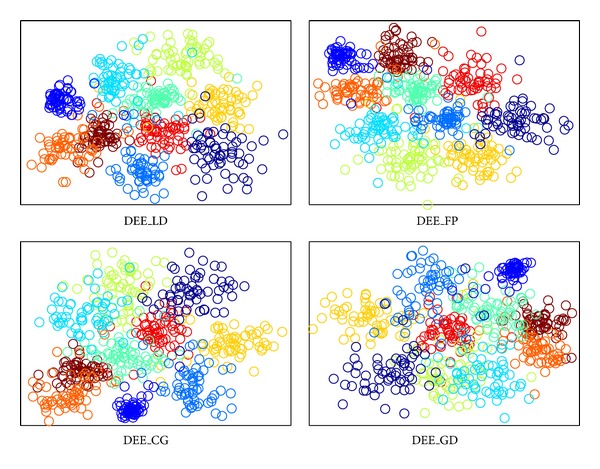
The clustering visualization for USPS data set with different training methods.

**Figure 6:**
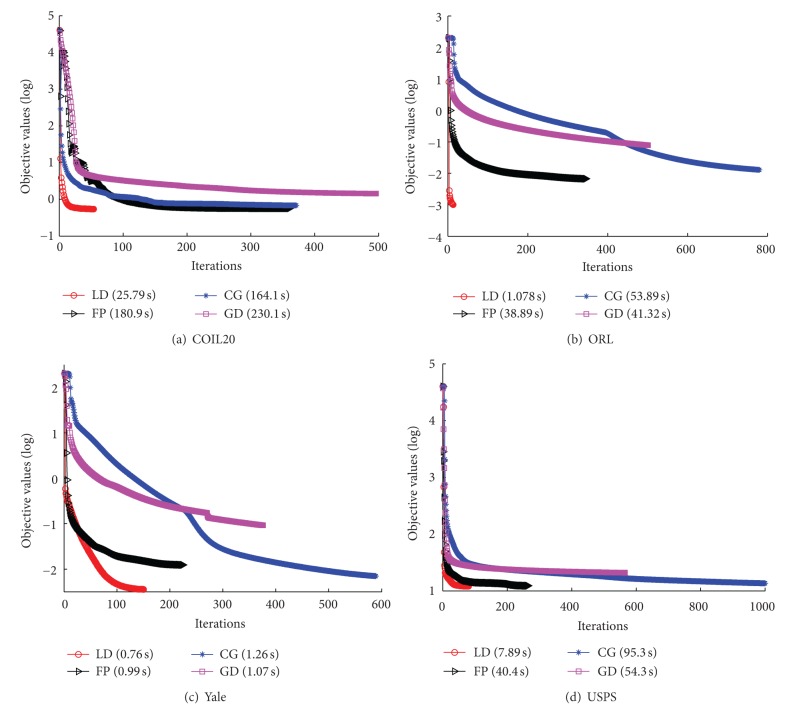
The objective values update versus progressive iterations with different training algorithms.

The visualization results are shown in Figures 2, 3, 4, and 5, where all the input data are projected into 2D space. The different colors stand for diverse classes.  Figure 6 demonstrates the learning curves as a function of progressive iterations. It also states the elapsed time in seconds for a single model construction. From Figures 2–5, we can see that, with different training methods, DEE is useful for clustering diverse class data. However, the LD method is clearly more competitive than the other three methods. In Figures 2 and 5, the colored coordinates show that DEE with LD method accurately separates the underlying disjoint structure present in diverse class. However, the other three methods have more overlaps incurred between different classes. In Figures 3 and 4, although all the four methods exhibit clearly the disjoint factors between diverse classes, the within class coordinates for FP, CG, and GD are more scattered, which is suboptimal for classification. From Figure 6, it is clear that DEE with LD method can achieve more precise objective values with less iteration times. In decreasing efficiency, the four methods should be roughly ordered as LD > FP > CG and GD. The CG method needs the most iteration times to meet the convergence condition. However, the objective value of CG is a little more precise than GD's value. This also explains why the clustering results for CG are slightly more accurate than GD's in Figures 2–5. Mostly, FP is more efficient than CG and GD, and it costs less time for completing a DEE model construction. Nevertheless, the runtime in every iteration loop for FP is more than CG and GD. So the construction time for FP is slower than GD in the COIL20 dataset, where these two methods spend close iteration times. LD not only achieves the most precise objective values, but also requires the least runtime. Take ORL dataset as example; LD needs only 13 iterations to obtain the optimal objective value, but FP needs about 390 iterations for the same convergence condition. So, LD costs about 1 second to construct the DEE model, which is about 38 times faster than FP, the second efficient method in the order. This result adheres to the theoretical analysis in Section 2 that the spectral direction is more useful for rapid convergence.

### 4.3. Evaluation of Different Embedding Algorithms

**Figure 7:**
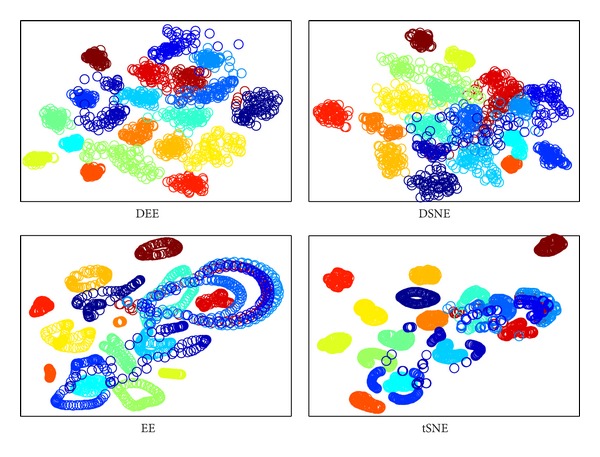
The clustering visualization for COIL20 data set with different embedding algorithms.

**Figure 8:**
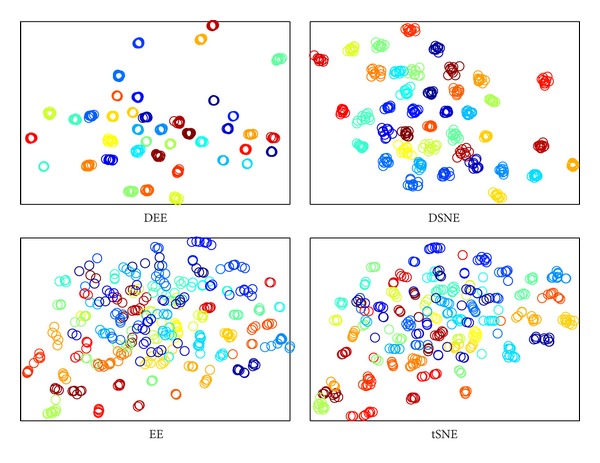
The clustering visualization for ORL data set with different embedding algorithms.

**Figure 9:**
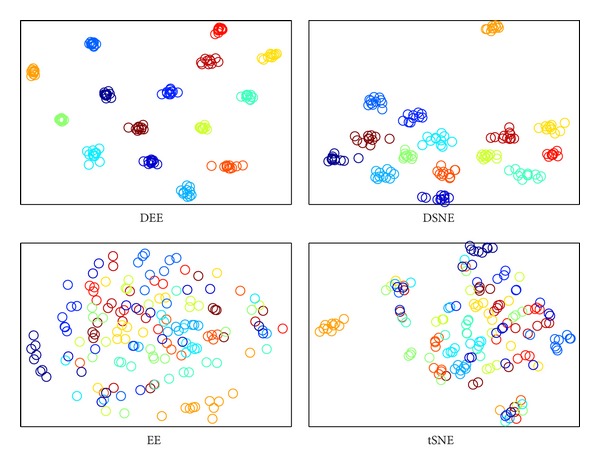
The clustering visualization for Yale data set with different embedding algorithms.

**Figure 10:**
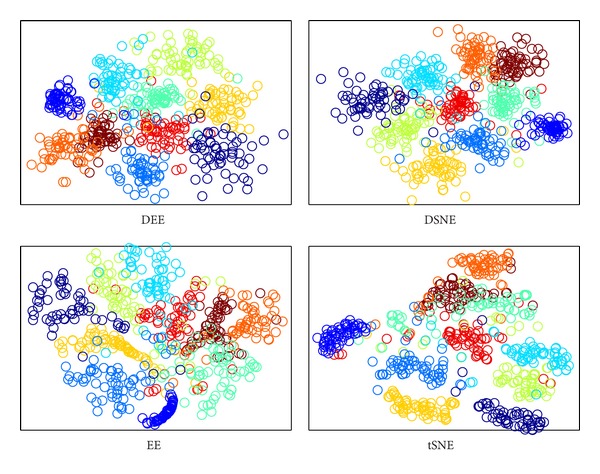
The clustering visualization for USPS data set with different embedding algorithms.

The DEE model with LD strategy was proved to be the most effective and efficient method in the preceding evaluation. To begin the classification performance analysis of the proposed approach, we secondly compare it with other embedding algorithms for assessing its capability of avoiding overlaps with different classes. We carried out comparisons to DSNE, EE, and *t*-SNE in 2D embedding space. The visualization results are illustrated in Figures 7, 8, 9, and 10. What is clear from these figures is that DEE and DSNE are more capable of separating data apart from different classes than EE and *t*-SNE. Note that EE and *t*-SNE both neglect the class labels for model construction. This demonstrates that the class labels ought to be a far more significant factor for enhancing classification performance. Furthermore, from Figures 7 and 9, we can see that DSNE not only has more interclass overlaps, but also has more intraclass scatters than DEE. This is due to two main factors. First, the traditional SNE-type embedding algorithms such as *t*-SNE or DSNE use normalization probabilities, which is cumbersome and unnecessary. However, DEE abandons the normalization term but focuses on the important and explicit relation between nonlinear and spectral methods, which makes DEE more robust to various types of data. Second, DEE uses the spectral direction for optimization, which is efficient and has no parameters to tune. Although DSNE uses conjugate gradient method for optimization, there are many parameters that need to be manually adjusted, which is difficult and time consuming. Besides, the conjugate gradient method is apt to fall into local optimum, which leads to cluttered subspace coordinates.

### 4.4. The Evaluation of Classification Performance

**Figure 11:**
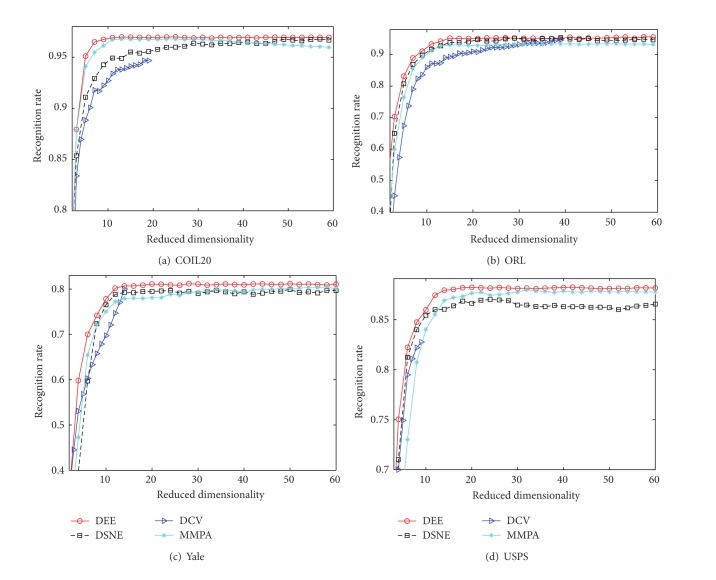
Recognition rate versus subspace dimension on different datasets.

In [19, 30], some comparison studies of SNE-type embedding algorithms and spectral methods were demonstrated for image data and hyperspectral data, respectively. Those demonstrations showed that, while SNE-type embedding algorithms do improve the classification performance, the requirement of even more concise subspace dimension remains a challenge. From the experimental results in Section 4.3, we know that the class labels are important to the classification performance. Here we compare DEE with three other supervised dimensionality reduction techniques, DSNE, DCV, and MMPA. DCV and MMPA are two recently proposed spectral methods. DCV has no special parameters needed to be tuned. For MMPA, we set the parametric pair *ε*_*w*_ and *ε*_*b*_ to be the average intraclass and interclass Euclidean distances, respectively. For DSNE, we follow the parametric set as in [30]. To illustrate the classification performance in the projected spaces, a nearest neighbor classifier is used to produce the decision results. For COIL20 dataset, we randomly selected 15 samples for each object as the training samples. For ORL and Yale datasets, we uniformly provided 50% training samples. In USPS, 25 samples in each class were used for input data. All the rest data were used as testing samples.  Figure 11 shows the recognition rate versus progressive subspace dimension for DEE, DSNE, DCV, and MMPA in four different datasets. All the results in Figure 11 were come into being with ten replications. From this illustration, we can see that the maximum subspace dimension of DCV is limited to* C*-1, due to the rank of the difference matrix. This limitation makes DCV perform poorly in some datasets. Besides, DCV demands more null space information in intraclass scatter matrix for better recognition rate. So, in USPS dataset, the optimal accuracy for DCV is only 83%, which is the worst compared with other three algorithms. Without this restriction, the other three algorithms are free for the choice of subspace dimension. However, since the conjugate gradient method is unstable and suboptimal, DSNE only gets a little better recognition rate than DCV, and its accuracy curve is more fluctuant. The best recognition rate of MMPA and DEE is very close. By comparison, the recognition rate curve of DEE is smoother than MMPA's. This reduces the complexity of choosing a proper subspace dimension value in a wide range for the users. Furthermore, DEE reaches the higher recognition rate with lower subspace dimension value, which complies with the essence of dimensionality reduction. In other words, DEE is capable of using as little as possible subspace dimensions for representing the original feature space.

## 5. Conclusions

A new embedding algorithm based on EE is proposed in this paper. The algorithm can be used for clustering visualization and classification. Our experimental illustrations were focused on image data embedding; however, this algorithm can also be extended to dimension reduction of other data without any adjustment. DEE, as a supervised embedding algorithm, is capable of pulling together the intraclass examples as well as pushing away the interclass examples. This “pull-push” property makes DEE qualified for discrimination tasks. The main disadvantage of all the SNE-type algorithms is that their optimization is a nonconvex issue requiring relatively slow iterative process. We introduced the Laplacian search direction to improve this gradient based optimization strategy. Empirically, the solutions solved by Laplacian direction are faster and more effective than the existing optimization methods. The experimental results in this paper on four image datasets show that DEE outperforms existing state-of-the-art algorithms for clustering visualization and classification. With fewest computation cost and more concise subspace dimension, DEE shows better embedded structure and reaches highest recognition rate.

In future work, we plan to speed up the computation cost in every iteration loop for LD strategy, which brings “big data” within reach of visualization and classification. We will also investigate the scalable optimization of all SNE-type algorithms, from which we can establish the uniform SNE based embedding framework.
